# UV-Light-Tunable p-/n-Type Chemiresistive Gas Sensors Based on Quasi-1D TiS_3_ Nanoribbons: Detection of Isopropanol at ppm Concentrations

**DOI:** 10.3390/s22249815

**Published:** 2022-12-14

**Authors:** Victor V. Sysoev, Andrey V. Lashkov, Alexey Lipatov, Ilya A. Plugin, Michael Bruns, Dirk Fuchs, Alexey S. Varezhnikov, Mustahsin Adib, Martin Sommer, Alexander Sinitskii

**Affiliations:** 1 Department of Physics, Yuri Gagarin State Technical University of Saratov, 410054 Saratov, Russia; 2 Center for Probe Microscopy and Nanotechnology, National Research University of Electronic Technology, 124498 Moscow, Russia; 3 Department of Chemistry, Biology & Health Sciences, South Dakota School of Mines and Technology, 501 E. Saint Joseph St., Rapid City, SD 57701, USA; 4 Institute for Applied Materials and Karlsruhe Nano Micro Facility, Karlsruhe Institute of Technology (KIT), Hermann-von-Helmholtz-Platz 1, 76344 Eggenstein-Leopoldshafen, Germany; 5 Institute for Quantum Materials and Technologies, Karlsruhe Institute of Technology (KIT), Hermann-von-Helmholtz-Platz 1, 76344 Eggenstein-Leopoldshafen, Germany; 6 Institute for Microstructure Technology, Karlsruhe Institute of Technology (KIT), 76344 Eggenstein-Leopoldshafen, Germany; 7 Department of Chemistry, University of Nebraska—Lincoln, Lincoln, NE 68588, USA

**Keywords:** gas sensor, multisensor array, alcohol, room temperature, UV radiation, benzene, titanium trisulfide

## Abstract

The growing demand of society for gas sensors for energy-efficient environmental sensing stimulates studies of new electronic materials. Here, we investigated quasi-one-dimensional titanium trisulfide (TiS_3_) crystals for possible applications in chemiresistors and on-chip multisensor arrays. TiS_3_ nanoribbons were placed as a mat over a multielectrode chip to form an array of chemiresistive gas sensors. These sensors were exposed to isopropanol as a model analyte, which was mixed with air at low concentrations of 1–100 ppm that are below the Occupational Safety and Health Administration (OSHA) permissible exposure limit. The tests were performed at room temperature (RT), as well as with heating up to 110 °C, and under an ultraviolet (UV) radiation at λ = 345 nm. We found that the RT/UV conditions result in a n-type chemiresistive response to isopropanol, which seems to be governed by its redox reactions with chemisorbed oxygen species. In contrast, the RT conditions without a UV exposure produced a p-type response that is possibly caused by the enhancement of the electron transport scattering due to the analyte adsorption. By analyzing the vector signal from the entire on-chip multisensor array, we could distinguish isopropanol from benzene, both of which produced similar responses on individual sensors. We found that the heating up to 110 °C reduces both the sensitivity and selectivity of the sensor array.

## 1. Introduction

One-dimensional (1D) nanostructures, high-aspect-ratio objects where at least one dimension does not exceed 100 nm, are widely recognized as extremely sensitive material platforms for gas sensors [1,2,3,4,5,6]. The research on one-dimensional nanostructures, including semiconductor nanowires (NWs), nanobelts, and carbon nanotubes, revealed a multitude of size-dependent electronic, magnetic, optical, and chemical properties that can benefit many sensing applications. Since one-dimensional nanostructures have a large surface-to-volume ratio, a significant fraction of their atoms are exposed to analyte species [7]. Another major advantage of one-dimensional nanostructures related to their morphology/shape is that the Debye length, λ*_D_*, a measure of penetration of the surface electric field into the bulk, for many semiconductor NWs, is comparable to their radius [8]. As a result, the electronic properties of NWs are strongly affected by the surface processes, such as doping and chemical reactions [9]. In particular, binding of analytes to the surface of a sensor material results in depletion or accumulation of carriers in the bulk of a NW and dramatically changes its electrical conductivity. These reasons make a sound basis for the NW-based gas sensors that are capable of detecting gases of interest down to ppb levels even under environmental conditions [10,11].

More recently, the attention of the materials science community has been drawn to the large class of two-dimensional (2D) materials, such as graphene and transition metal dichalcogenides [12]. These materials are typically prepared through exfoliation of layered van der Waals (vdW) crystals [12] or by the direct growth in a two-dimensional form [13]. Two-dimensional materials also provide a multitude of interesting physical and chemical properties and have a planar structure, where a large number, if not all, of atoms are exposed to the environment. These materials combine rather large lateral dimensions with atomically thin thickness, which makes their properties largely comparable to the one-dimensional materials. In particular, chemiresistive gas sensors based on two-dimensional materials became a large and rapidly developing area of research, and many such sensors based on graphene [14], graphene oxide [15], molybdenum disulfide (MoS_2_) [16], MXenes [17,18], and other two-dimensional materials have been demonstrated in recent years [19,20]. However, unlike the one-dimensional structures, in which an electric field generated by an adsorbed analyte molecule can penetrate a thin NW entirely and largely affect its electrical conductivity, it would have only a local effect on the electronic transport through a micrometer-scale flake of a two-dimensional material, whose lateral dimensions would be much larger than λ*_D_*. Therefore, decreasing lateral dimensions of a conducting channel of a sensor based on a two-dimensional material to essentially move towards a one-dimensional regime could be a strategy to improve the sensor’s sensitivity.

In light of these considerations, when comparing different layered vdW crystals, titanium trisulfide (TiS_3_), a semiconductor material with a bandgap of ~1 eV [21,22], might be considered as a promising material for gas sensor applications. Similar to very extensively studied transition metal dichalcogenides (TMDs), such as MoS_2_, MoSe_2_, WS_2_ and WSe_2_ [23,24], the titanium trisulfide crystals have a layered structure with weak vdW bonding between layers [25]. However, unlike layers of TMDs which exhibit a strong in-plane covalent bonding, the layers of TiS_3_ consist of one-dimensional chains that also have weak vdW-like interactions between them [26]. Therefore, mechanical exfoliation of bulk TiS_3_ crystals typically results not in randomly shaped two-dimensional flakes, as in case of TMDs, but in high-aspect-ratio nanoribbons [26,27,28], which can be viewed as semiconducting NWs. Furthermore, TiS_3_ is widely recognized as a very promising semiconductor material with high theoretically predicted mobility [29,30], highly anisotropic electronic and optoelectronic properties [31,32], low contact resistances with conventional contact metals [33], and intriguing low-temperature physics [34]. The remarkable electronic characteristics of TiS_3_ crystals justify their investigation for gas sensing applications.

A number of successful gas sensor designs based on one-dimensional materials employed random networks of NWs that were drop-casted on pre-patterned electrodes from a solution [35,36]. Therefore, we have considered this approach to test the chemiresistive properties of TiS_3_ NWs grown via a high-temperature reaction between titanium and sulfur in vacuum according to the previously developed protocols [37,38,39,40]. Herein, we report on chemiresistive properties of TiS_3_ NWs upon their exposure to isopropanol, which was used as a model analyte, mixed with synthetic air at part-per-million (ppm) concentrations. Isopropanol is widely used in everyday life, but could be toxic at high concentrations, resulting in poisoning, respiratory problems, eye irritation, and allergic skin reaction [41,42]. Therefore, the development of gas sensors for a reliable detection of isopropanol is an important technological problem.

## 2. Materials and Methods

**Figure 1:**
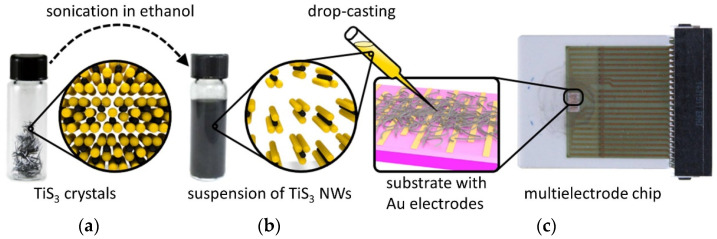
Fabrication of chemiresistive gas sensors based on TiS_3_ NWs. (**a**) Optical photograph of as-grown TiS_3_ crystals. The scheme shows a fragment of the crystal structure of TiS_3_. Yellow spheres—sulfur; black spheres—titanium. (**b**) Optical photograph of the dispersion of TiS_3_ crystals in ethanol. The scheme shows 1D TiS_3_ chains that were exfoliated from bulk crystals by ultrasonication. (**c**) Optical photograph of a multielectrode sensor chip. The scheme shows the drop-casting of the TiS_3_ suspension on the electrode area of the chip.

To prepare the TiS_3_ NWs, we used a 0.25-mm-thick titanium foil (99.99+%, Alfa Aesar, Haverhill, MA, USA) and a sulfur powder (analytical grade, Sigma-Aldrich, St. Louis, MO, USA), which was taken in a 10% excess to the Ti:S stoichiometric ratio of 1:3 to ensure the absence of TiS_2_ byproduct. Both reactants were combined in a quartz ampoule and sealed under a vacuum of about 0.2 Torr. The ampoule was placed in a tube furnace in a way that it had a small temperature gradient and annealed at about 550 °C for 5 days. The procedure resulted in the formation of needle-like TiS_3_ crystals on the Ti foil and on the surface of the quartz ampoule, which was closer to the hotter part of the tube furnace. Upon cooling the furnace, the excess sulfur accumulated on the opposite side of the ampule. The details on the growth and materials characterization of TiS_3_ whiskers can be found in our previous works [26,28]. Figure 1a shows the optical photograph of needle-like TiS_3_ crystals along with a scheme of the crystal structure of TiS_3_. These crystals were dispersed in ethanol (99.8%, Sigma-Aldrich), which was first dried using molecular sieves to reduce the water content, and then subjected to ultrasonication. As we extensively discussed in our previous works, the basic building blocks of TiS_3_ crystals are 1D chains that are assembled into a three-dimensional crystal structure through weak interactions [26,28]. It was previously shown that these 1D chains could be easily separated from each other by mechanical exfoliation, which splits bulk TiS_3_ crystals into thin nanoribbons with their long axes corresponding to the direction of 1D chains [26]. Similarly, the ultrasonication-assisted exfoliation of TiS_3_ crystals splits them along the 1D chain direction, as shown schematically in the inset in Figure 1b. The resulting suspension (Figure 1b) was collected by a pipette dispenser, drop-casted on the surface of a multielectrode chip, as drawn in the scheme in Figure 1c, and dried in ambient conditions. The density of the TiS_3_ nanowires in the drop-casted film was adjusted to yield the output resistance in the kOhm–MOhm range, which was appropriate for the electronics utilized in this study.

The multielectrode sensor chip was fabricated on an alumina substrate, which was equipped with 17 coplanar electrodes (50 μm × 4 mm) with a spacing of 50 μm [43]. The front side of the chip also contained two meander-shaped thermoresistors. The metallization of the chip was performed by Au printing; the thickness of the gold layers was about 10 μm. Each pair of adjacent electrodes and the chip area in between constituted an individual chemiresistive sensor. Thus, the entire chip contained 16 sensors as a multisensor array. The electrodes and the thermoresistors were routed to a multi-pin socket (Erni Electronics, Adelberg, Germany) for connecting to the readout electronics of a KAMINA unit [44]. The optical photograph of the chip is shown in Figure 1c. Similar chips were previously successfully employed in studies of a great variety of nanomaterials, such as graphene [14], graphene oxide [15,45], graphene nanoribbons [46,47], and MXenes [18].

TiS_3_ NWs were chemically characterized by X-ray photoelectron spectroscopy (XPS). For this purpose, the nanowires were immobilized onto Au-coated silicon substrates, and the XPS measurements were performed using a K-Alpha^+^ XPS spectrometer (Thermo Fisher Scientific, East Grinstead, UK). The details of the data acquisition and processing via the Thermo Avantage software are described elsewhere [48]. All samples were analyzed using a microfocused, monochromated Al Kα X-ray source with a 400 µm spot size. The spectra were fitted with one or more Voigt profiles. For intense peaks and/or peaks clearly evidenced by the peak shape, the binding energy uncertainty was around ±0.1 eV. In case of weak peaks and no direct justification by the peak shape, the uncertainty was set to ±0.2 eV. The analyzer transmission function, Scofield sensitivity factors [49], and effective attenuation lengths (EALs) for photoelectrons were applied for quantification. EALs were calculated using the standard TPP-2M formalism [50]. All spectra were referenced to the C1s peak in hydrocarbons at 285.0 eV binding energy [51], controlled by means of the well-known photoelectron peaks of metallic Cu, Ag, and Au.

The crystal structure of the TiS_3_ NWs was evaluated via X-ray diffraction (XRD) analysis using a two-circle diffractometer (Stadi P, STOE Co., Darmstadt, Germany) in the Bragg-Brentano geometry, equipped with a Ge (111) monochromator and using MoK_α_ radiation, λ = 0.70926 Å [52].

The transmission electron microscopy (TEM) images were acquired using a Tecnai Osiris scanning transmission electron microscope (FEI, Hillsboro, OR, USA) equipped with a HAADF detector and a X-FEG high brightness Schottky field emission gun. The accelerating voltage was 200 kV. The TiS_3_ NWs were visualized on lacey-carbon-covered 300-mesh copper grids.

The TiS_3_ NW-based chip was inspected with an optical microscope at ×7.5–×500 magnifications. The morphology of the TiS_3_ layer on the chip was also visualized by scanning electron microscopy (SEM, Supra 60VP, Carl Zeiss AG, Jena, Germany) at the accelerating voltage of 5.0 kV.

**Figure 2:**
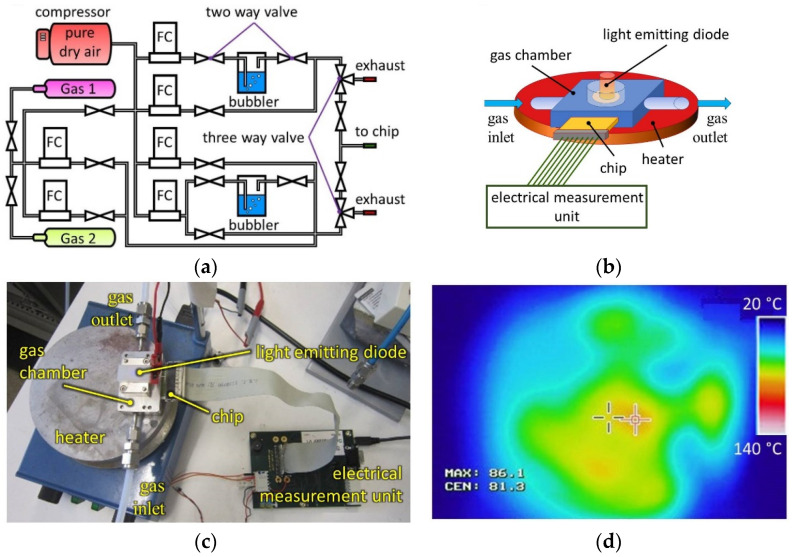
Chemiresistive gas–sensor experimental setup to study TiS_3_ NWs placed on a multielectrode chip. (**a**) Scheme of the gas delivery system that can supply two analytes (Gas 1 and Gas 2) at controlled concentrations. FC—flow controller. (**b**) Scheme of the entire gas sensor setup, which includes a housing chamber, a UV light emitting diode (LED), an external heater, and an electrical measurement unit. (**c**) Photograph of the gas sensor setup connected to KAMINA electronics (not shown). (**d**) Infrared image of the heated support of the chip chamber for temperature measurements.

The gas sensing experiments were carried out using a homemade gas delivery setup that is schematically shown in Figure 2a. It consists of a number of gas lines that were fed primarily by synthetic air (79 vol. % N_2_, 21 vol. % O_2_) from a calibrated source. This carrier gas was mixed with isopropanol or benzene vapors sourced from calibrated mixtures with synthetic air (250 ppm) to prepare the test mixtures containing 1-100 ppm concentrations of the analyte. The reported measurements were performed in a flow mode in dry air background. The entire setup was managed by custom software using a personal computer (PC). The TiS_3_ NW-based chip was measured in a stainless-steel gas chamber with inlet and outlet tubes for gas flow (Figure 2b). The front side of the chamber had a quartz window, through which the chip could be illuminated using an ultraviolet (UV) light emitting diode (LED), λ = 345 nm [53]. The UV-LED was powered at 0.05 W using a programmable power supply unit (PN 300, Grundig Digimess, Neu-Isenburg, Germany). For measurements at elevated temperatures, we placed the gas chamber with a TiS_3_ NW-based chip on a hotplate (Ikamag RCT, IKA Labortechnik, Janke & Kunkel GmbH & Co., Staufen im Breisgau, Germany), as shown in Figure 2c. The operating temperature was monitored with the chip thermoresistors, which were calibrated using an infrared (IR) camera (MikroSHOT 9010738, Mikron Infrared Inc., Oakland, NJ, USA). A typical IR image of the hotplate under operation is given in Figure 2d.

During the measurements, the gas flow through the chip was maintained at 500 sccm by high-precision mass flow controllers (FC, MKS Instruments Deutschland GmbH, Munich, Germany). The chip was exposed to the analyte mixtures for 60 min and then purged with air for 120 min. These time intervals were chosen from preliminary studies to not only measure the sensor response but also to collect enough data for vector signals from the on-chip multisensor array, which were later analyzed by a pattern recognition technique. We have tested the gas-sensing properties of TiS_3_ NWs at room temperature (RT), as well as at elevated temperatures of 60 ± 4 °C and 110 ± 5 °C, with and without UV-LED irradiation. The uncertainties indicated for the elevated temperatures were primarily caused by the temperature fluctuations of the hotplate. Higher temperatures were not tested to avoid oxidation of TiS_3_ nanowires.

The direct current (dc) electrical conductance of the TiS_3_ NW sensing elements was read out by PC-managed KAMINA unit electronics with a multiplexing card at a rate of about 1 Hz for the entire array. The minor variations/spikes in the sensor resistances appeared primarily due to the fluctuating operation of the hotplate and were smoothed using the Savitzky-Golay filtration.

The vector signals generated by the on-chip multisensor array toward analytes were processed with linear discriminant analysis (LDA) [54]. The technique transforms a *N*-dimensional raw signal to a reduced (*N* − 1) space, where *N* is the number of input classes for recognition, to maximize the ratio between inter-class and in-class variations. In addition to class recognition, this method allows a visualization of the results in a comprehensive manner that is intuitively understandable by a user, which provides an advantage over other artificial intelligence techniques. The axes in the LDA coordinate system, called LDA components, indicate features of the vector signals related to the differentiation of the specific input classes.

## 3. Results and Discussion

### 3.1. Physical Characterization of TiS_3_ NWs

**Figure 3:**
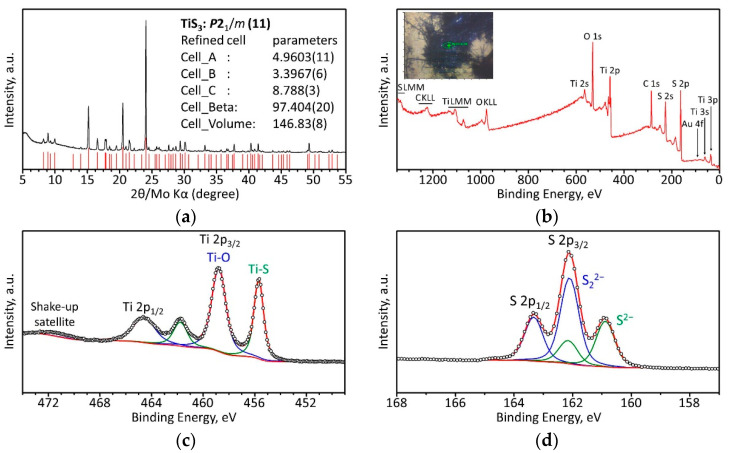
Characterization of TiS_3_ NWs by means of XRD and XPS techniques. (**a**) XRD pattern of a TiS_3_ powder. (**b**) XPS survey spectrum taken on the TiS_3_ material deposited on an Au-coated Si substrate. The absence of Au-related photoelectron peaks confirms the high density of TiS_3_ NWs on the substrate, as can be seen in the optical photograph in the inset (black needles are TiS_3_ NWs on an Au-coated Si substrate, green spot shows the area where the XPS spectra were recorded). (**c**) Ti 2p and (**d**) S 2p XPS spectra of TiS_3_ NWs. In panels (**c**,**d**), the open circles are the experimental data, the red curves are the respective envelopes of all peaks, and the green and blue curves are the fitted doublets of the assigned components.

To confirm the formation of TiS_3_ and verify its purity, we characterized the synthesized material using X-ray diffraction (XRD) analysis. The resulting XRD pattern of the powdered sample of TiS_3_ NWs is shown in Figure 3a and demonstrates sharp and intense peaks, indicating high crystallinity of the sample. All reflections of the XRD pattern were indexed using the *P*2_1_/*m* space group and the arrangement of atoms corresponding to the ZrSe_3_-type structure [26,28]. No other phases were present, confirming the high purity of the sample. The unit cell parameters were found to be *a* = 4.9603(11) Å, *b* = 3.3967(6), *c* = 8.788 Å, and *β* = 97.40(2)°, which are in line with previous experiments [26,28,55].

XPS measurements were performed in order to obtain information on chemical binding states. The spectra were collected on TiS_3_ NWs that were deposited on an Au-coated Si substrate, see the optical photograph in the inset in Figure 3b. The XPS survey spectrum in Figure 3b further proves the purity of the TiS_3_ crystals, which is in agreement with the XRD data. In addition, the absence of Au-related photoelectron peaks in the spectrum (Figure 3b) suggests a dense coverage of the Au-coated Si substrate with TiS_3_ NWs. Figure 3c,d show the high-resolution XPS spectra of the Ti 2p and S 2p regions, respectively. The binding energies of Ti 2p_3/2_ = 455.7 eV, S 2p_3/2_ = 160.9 eV (S^2−^), and S 2p_3/2_ = 162.1 eV (S_2_^2−^) measured for the TiS_3_ crystals are in a good agreement with data reported in the literature [33,56]. Additionally found photoelectron peak at Ti 2p_3/2_ = 458.8 eV and the corresponding O 1s peak at 530.3 eV (not shown) were attributed to TiO_2_ and Ti oxysulfide species, correspondingly [56,57,58], suggesting a slight oxidation of the samples at the surface of the TiS_3_ NWs [56,59]. Taking the atomic concentrations derived from the Ti 2p and S 2p peaks assigned to TiS_3_ NWs, we could estimate the near-surface stoichiometry, [S]/[Ti], to be 3.9 ± 0.1, which exceeds the expected stoichiometry of 3:1. Such a discrepancy is explained by the surface Ti oxysulfide species which were not accounted in the [S]/[Ti] stoichiometry calculation.

**Figure 4:**
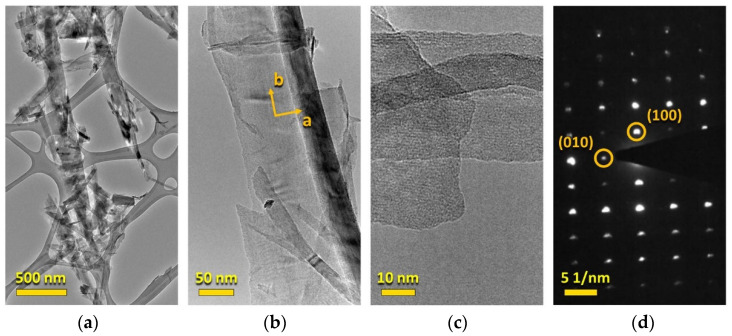
TEM analysis of solution-exfoliated TiS_3_ nanosheets on a lacey-carbon Cu grid. (**a**–**c**) TEM images at different magnifications. (**d**) SAED pattern of a representative TiS_3_ crystal.

The solution-exfoliated TiS_3_ NWs were studied by TEM. The sample was prepared by depositing the TiS_3_ NW suspension on a TEM grid and drying it in air. The TEM analysis revealed the presence of nanowires (Figure 4a) and thin flakes of TiS_3_ (Figure 4a,b), which also had elongated shapes. These TEM images demonstrate that ultrasonication in ethanol results in very thin TiS_3_ NWs with high surface area favorable for sensor applications. The selected area electron diffraction (SAED) image of a typical TiS_3_ crystal (Figure 4d) shows distinct reflections, which further confirm the high crystallinity of the sample. The analysis of the SAED pattern reveals the presence of (100) and (010) reflections, indicating that the crystals primarily exfoliated perpendicularly to the *c*-axis of the TiS_3_ structure.

**Figure 5:**
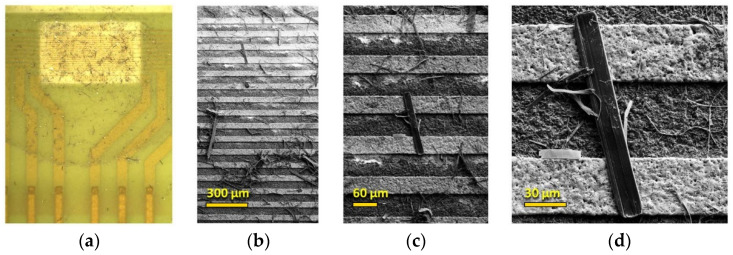
Microscopic characterization of the TiS_3_ NW mat deposited on the multielectrode chip. (**a**) Optical photograph of the chip surface. (**b**–**d**) SEM images of the NW mat on a substrate with gold electrodes (bright horizontal stripes) at various magnifications.

Following the drop-casting of the TiS_3_ NWs on the chip, we examined the sensor devices by optical and electron microscopies. The resultant TiS_3_ layer had a good adhesion and sufficient mechanical stability. Typical microscopic images taken at various magnifications are shown in Figure 5.

As expected, the nanowires with different shapes and diameters are randomly distributed on a substrate as a stochastic mat. Some large NWs are long enough to entirely bridge the 50 µm inter-electrode distance (Figure 5d), while many smaller TiS_3_ nanoribbons build a network composing the mat layer. The nonuniformity of the mat in terms of the size, density, and orientation of TiS_3_ NWs in different areas of the chip translates into a variation of the electronic properties of different sensor elements. This device-to-device variation in the on-chip sensor array is important for the electronic nose concept of analyte recognition (see, for instance, [14,15]), as discussed in Section 3.2.

### 3.2. Gas-Sensing Performance of TiS_3_ NWs

**Figure 6:**
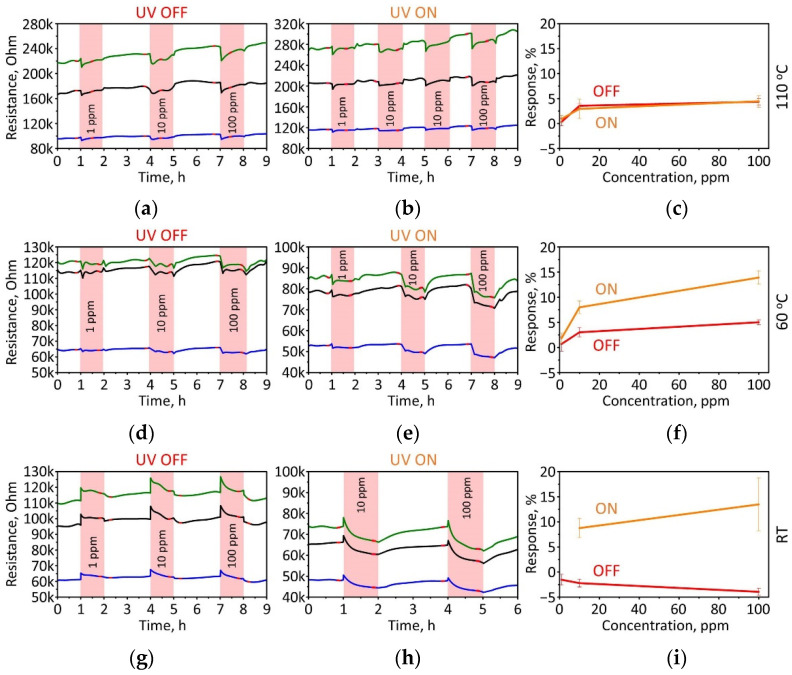
The chemiresistive response of TiS_3_ NW mat-based sensor elements upon their exposure to isopropanol vapors. (**a**,**b**) R(t) transients at 110 °C without (**a**) and with (**b**) UV-LED radiation. (**d**,**e**) R(t) transients at 60 °C without (**d**) and with (**e**) UV-LED radiation. (**g**,**h**) R(t) transients at RT without (**g**) and with (**h**) UV-LED radiation. The red data points were used for response calculations. (**c**,**f**,**i**) Response-to-concentration curves at various operation regimes: 110 °C (**c**), 60 °C (**f**), RT (**i**). The error bars represent the scattering observed over the sensor array. The green, black and blue curves indicate resistances of three different sensor elements.

Figure 6 shows the resistance changes of three typical TiS_3_ NW sensor elements upon their exposure to isopropanol vapors mixed with synthetic air at concentrations varying from 1 to 100 ppm. The gas sensing performance was tested at three different temperatures (RT, 60 °C, 110 °C) with and without UV radiation. In all regimes except for RT with UV turned off, the exposure to isopropanol vapor results in a reproducible reduction of the sensor resistance. A similar effect was observed in sensors based on titanium disulfide nanosheets when the resistivity of the sensor channel decreased upon exposure to vapors of reducing gas, H_2_S, at room temperature [60]. The resistance variation can be expressed using gas response (*S*) according to the formula

(1) $$S=\left(\frac{R_{a}}{R_{g}}-1\right)\times 100\%,$$

where *R_a_* is the resistance of the sensor element when exposed to analyte/air mixture and *R_g_* is the resistance of the sensor element in background air.

The largest variation of resistance was observed when the sensor array was measured at room temperature with UV activation (i.e., RT/UV), with the response to 100 ppm of isopropanol reaching 13.4 ± 5.3%. The scattering in the results accounts for the device-to-device variation in the on-chip array. A similar response was observed under UV irradiation at 60 °C with *S* = 13.9 ± 1.3%. A higher temperature of 110 °C diminishes the effect of the UV, as the observed resistance variation upon exposure to vapors at 100 ppm concentration results in a response of only 4.5 ± 1.2%. It is worth noting that the measured concentrations are below the permissible exposure limit established by the United States Occupational Safety and Health Administration (OSHA) regulations of 400 ppm averaged over an eight-hour work shift [61].

The dependence of the gas sensor response on the isopropanol concentration, UV exposure, and temperature is shown in Figure 6c,f,i. A higher concentration of the analyte results in a higher response for almost all studied conditions except for RT/UV Off. This behavior is consistent with the XPS results that demonstrated a partial oxidation of the surface of TiS_3_ NWs in ambient conditions, implying the presence of the oxygen adspecies. According to data reported in Ref. [59], the adsorbed oxygen could acquire a free electron from a NW and become a negatively charged ion, as shown in Equation (2)

(2) $$O_{2}+e^{-}\to O_{2}^{-}.$$

Here, we suggest that O^−^ and/or O^2−^ adspecies would not appear at low temperatures even under UV activation, as discussed in numerous works on metal oxide-based gas sensor elements [62]. However, we should clarify what type of oxygen ions is primarily adsorbed on the TiS_3_ surface. Accounting for the n-type conductivity of TiS_3_ [63], the oxygen adsorption enhances its resistance in the air environment. The adsorbed species facilitate the appearance of surface defects that lead to electron carrier scattering and lower carrier mobility, resulting in a higher resistance, as frequently discussed in the literature on low-dimensional structures, including two-dimensional materials [64].

When the sensor is exposed to isopropanol vapor, the analyte molecules may interact not only with TiS_3_ but also with the oxygen adspecies, especially under the conditions of UV radiation and/or heating. This can be illustrated by the following reaction, which demonstrates the general pathway of the process and the most anticipated products rather than a specific stoichiometric reaction mechanism:

(3) $$2C_{3}H_{7}\mathrm{OH}+9O_{2}^{-}\to 6{\mathrm{CO}}_{2}↑+8H_{2}O↑+9e^{-}.$$

The UV energy and elevated temperatures support this redox reaction. As a result, the oxygen adspecies are scavenged from the surface of TiS_3_, while the gaseous products are removed from the system. In addition to the removal of surface defects, this reaction will also increase the free electron concentration in TiS_3_ NWs. Altogether, the NW resistance drops down, as observed in the experiment. A rather slight change in resistance is reasoned by a relatively high concentration of electrons primarily available in TiS_3_. Interestingly, a tunable n-/p-type electronic behavior, which was highly dependent on the surface oxidation and oxygen adspecies, was recently demonstrated for HfS_3_ that is isostructural and has many similar properties to TiS_3_ [65].

It is worth noting that the necessity of UV assistance and heating, at least to 60 °C, to support the chemiresistive effect in TiS_3_ NWs is evidenced by observations of resistance behavior and the gas response at RT without UV-LED radiation (Figure 6g,i). Indeed, we see that exposure of the material to isopropanol vapors yields a slight enhancement of the NW mat resistance, which is opposite to the effect described above. Similar behavior of the resistance and change in the gas response sign is frequently observed in various materials, such as metal oxides [66,67] and sulfides [68].

We suggest that the isopropanol molecules adsorb on the TiS_3_ surface and create more defect centers similar to oxygen species and further reduce the electron mobility. However, this effect is less pronounced than the one related to changing a free carrier concentration.

The data shown in Figure 6c demonstrate that increasing the temperature up to about 110 °C limits the effect of UV activation. The observed gas response values become unaffected by the UV irradiation and decrease to the values measured at 60 °C with UV turned off. This phenomenon might be related to enhancing phonon oscillations in the crystal lattice of TiS_3_ NWs, which does not allow the isopropanol molecules to effectively adsorb on the surface of TiS_3_ and interact with oxygen adspecies.

**Figure 7:**
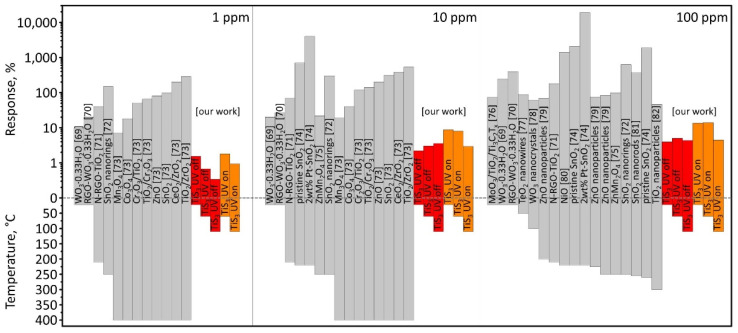
Comparison of experimentally observed chemiresistive responses of various materials to isopropanol at 1–100 ppm concentrations at different operating temperatures. The data are extracted from the literature [69,70,71,72,73,74,75,76,77,78,79,80,81,82] and compared to the results for TiS_3_ NW mat-based sensor elements. (1) Responses to 1 ppm of isopropanol. (2) Responses to 10 ppm of isopropanol. (3) Responses to 100 ppm of isopropanol.

We have compared our experimental chemiresistive responses to isopropanol vapors with similar recent reports on gas sensors based on metal oxides or their composites with two-dimensional structures [69,70,71,72,73,74,75,76,77,78,79,80,81,82]. The comparison charts are shown in Figure 7, where a two-axis diagram accounts for the response magnitude and the operating temperature when observing the response. Unlike TiS_3_ sensor studied in this work, the metal oxides need thermal activation by heating up to temperatures of 200 °C of above, which requires more energy when compared to the RT/UV regime for TiS_3_ NWs. Therefore, these structures could be considered in sensor applications where power consumption is a challenging task, such as for autonomous detection units.

In order to explore the possibility of selective detection of isopropanol, we exposed the TiS_3_ NW-based chip to benzene vapor, which is known to be more inert to interact with chemiresistive materials [83]. In the experiments utilizing the TiS_3_ NW sensors, we could only reliably register the chemiresistive response to 100 ppm concentration of benzene vapors.

**Figure 8:**
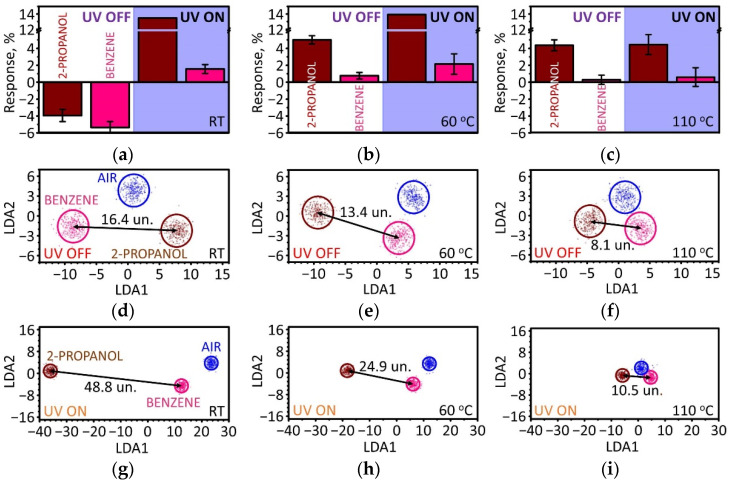
Comparison of chemiresistive responses of TiS_3_ NW mat-based sensor elements to isopropanol and benzene vapors, both at 100 ppm concentration. (**a**–**c**) Magnitudes of the chemiresistive responses at RT (**a**), 60 °C (**b**), and 110 °C (**c**) with and without UV assistance. (**d**–**i**) LDA diagrams of the vector signals from the on-chip sensor array under various operation regimes: RT (**d**), 60 °C (**e**), 110 °C (**f**), RT/UV (**g**), 60 °C/UV (**h**), and 110 °C/UV (**i**). The points are vector signals, ellipses are built according to a suggestion of normal distribution of data within each class around the gravity centers with 0.95 confidence level.

Figure 8a–c shows the response to benzene vapor at different conditions in comparison with the results obtained for 100 ppm of isopropanol vapor. At RT with no UV assistance, the responses to both analytes are comparable, of about 4–5%, and are of p-type, which agrees with our suggestion that the physisorbed molecules of both types increase scattering for the electron transport in the TiS_3_ NW mats. When UV is on, however, the benzene is not properly activated to interact with the surface oxygen adspecies, as in the case of isopropanol with the reaction (3). As a result, the response of *S* = 1.6% is lower than without UV, and when compared to the response to isopropanol. Still, the sign of the response is similar to isopropanol, making it difficult to distinguish between the analytes. It is still interesting to note that the UV activation at about 60 °C slightly improves the response to benzene, up to about 2.1%, with a further significant drop to about 0.6% at the UV/110 °C operation.

We tested selectivity of the analyte recognition by processing all gas sensing responses from the entire TiS_3_ NW-based sensor array using LDA algorithms. For this purpose, we analyzed the responses to isopropanol and benzene, both at 100 ppm concentration, and used the responses to synthetic air as a reference. The results of the LDA analysis are plotted in Figure 8d–i for various operation conditions. The points are the vector signals (resistance distributions) from the array while ellipses are built accounting for Gaussian distributions of in-class data around the corresponding gravity centers at 0.95 confidence level. In all cases, the clusters related to benzene and isopropanol are well distinguished from each other and from the air reference. Still, the Euclidian distances in the LDA space between the gravity centers of analyte-related clusters and the air-related cluster correlate with the response magnitudes, i.e., the signals to benzene, which shows weaker responses than isopropanol, are closer to the signals recorded in air. This behavior is similar to the chemiresistive response to isopropanol but at a smaller magnitude, which suggests the fundamental similarities of the interaction of TiS_3_ surface with the analytes under these operation conditions. Further insights into the origin of this effect and features related to various analytes are required [84].

In general, the TiS_3_ NW mat-based chip functioning without UV activation yields lower Euclidian distances in this LDA space between clusters related to two analytes, being 16.4 units (un.) at RT, 13.4 un. at 60 °C, and 8.1 un. at 110 °C. With UV assistance, the distances are much more pronounced, primarily along the first LDA component axis, and were found to be 48.8 un. at RT, 24.9 un. at 60 °C, and 10.5 un. at 110 °C. Therefore, the spatial variation of the NW mat density in the sensor array is sufficient upon fabrication to deliver the analyte-specific signals. Further selectivity improvement could be achieved for these sensor arrays using external impacts (see, for instance, [85]).

## 4. Conclusions

The pilot experiments to study a chemiresistive effect in the quasi-1D TiS_3_ NWs show that this material is suitable for gas sensors operating at room temperature towards detecting alcohol vapors in concentrations below the OSHA permissible exposure limit. The UV radiation of the nanowire surface by LED is shown to effectively facilitate the sensor response of n-type when the resistance drops upon exposure to isopropanol, in contrast to the p-type response observed at RT without UV. The reduction of the NW resistance might be well explained via redox reactions that occur at the surface in accordance with other research on sulfide materials. At the same time, the enhancement of NW resistance, up to about 14%, with the analyte under the RT conditions is very similar to effects observed in two-dimensional materials, such as graphene or MXenes, when the physisorbed molecules act as surface defects and result in electron scattering. Still, the n-type effect stimulated by UV irradiation was found to be more pronounced. Finally, we demonstrated the discrimination of two different analytes, isopropanol and benzene, using the multisensor approach and analyzing vector signals from all devices in the on-chip sensor array via pattern recognition algorithms, here LDA. The demonstrated approach for translating TiS_3_ crystals into NW-based gas sensor arrays with UV/temperature-tunable responses and a potential for analyte recognition could be applied to other quasi-one-dimensional transition metal trichalcogenides that exfoliate into one-dimensional crystals, such as HfS_3_ [65], ZrS_3_ [86], In_4_Se_3_ [87], and many others [21,34]. The results of this study may provide a promising avenue toward UV-activated energy-efficient gas sensors based on one-dimensional nanomaterials.

## Data Availability

The data presented in this study are available on request from the corresponding author.
